# Microbial interactions impact stress tolerance in a model oral community

**DOI:** 10.1128/spectrum.01005-24

**Published:** 2024-09-13

**Authors:** Gina R. Lewin, Emma R. Evans, Marvin Whiteley

**Affiliations:** 1 School of Biological Sciences and Center for Microbial Dynamics and Infection, Georgia Institute of Technology, Atlanta, Georgia, USA; 2 Emory-Children’s Cystic Fibrosis Center, Atlanta, Georgia, USA; University of Dundee, Dundee, United Kingdom

**Keywords:** *S. gordonii*, *A. actinomycetemcomitans*, polymicrobial interactions, disturbance, cross-feeding

## Abstract

Understanding the molecular mechanisms governing microbial interactions is crucial for unraveling the complexities of microbial communities and their ecological impacts. Here, we employed a two-species model system comprising the oral bacteria *Aggregatibacter actinomycetemcomitans* and *Streptococcus gordonii* to investigate how synergistic and antagonistic interactions between microbes impact their resilience to environmental change and invasion by other microbes. We used an *in vitro* colony biofilm model and focused on two *S*. *gordonii*-produced extracellular molecules, L-lactate and H_2_O_2_, which are known to impact fitness of this dual-species community. While the ability of *A. actinomycetemcomitans* to cross-feed on *S. gordonii*-produced L-lactate enhanced its fitness during co-culture, this function showed little impact on the ability of co-cultures to resist environmental change. In fact, the ability of *A. actinomycetemcomitans* to catabolize L-lactate may be detrimental in the presence of tetracycline, highlighting the complexity of interactions under antimicrobial stress. Furthermore, H_2_O_2_, known for its antimicrobial properties, had negative impacts on both species in our model system. However, H_2_O_2_ production by *S. gordonii* enhanced *A. actinomycetemcomitans* tolerance to tetracycline, suggesting a protective role under antibiotic pressure. Finally, *S. gordonii* significantly inhibited the bacterium *Serratia marcescens* from invading *in vitro* biofilms, but this inhibition was lost during co-culture with *A. actinomycetemcomitans* and in a murine abscess model, where *S. gordonii* actually promoted *S. marcescens* invasion. These data indicate that microbial interactions can impact fitness of a bacterial community upon exposure to stresses, but these impacts are highly environment dependent.

**IMPORTANCE:**

Microbial interactions are critical modulators of the emergence of microbial communities and their functions. However, how these interactions impact the fitness of microbes in established communities upon exposure to environmental stresses is poorly understood. Here, we utilized a two-species community consisting of *Aggregatibacter actinomycetemcomitans* and *Streptococcus gordonii* to examine the impact of synergistic and antagonistic interactions on microbial resilience to environmental fluctuations and susceptibility to microbial invasion. We focused on the *S. gordonii*-produced extracellular molecules, L-lactate and H_2_O_2_, which have been shown to mediate interactions between these two microbes. We discovered that seemingly beneficial functions, such as *A. actinomycetemcomitans* cross-feeding on *S. gordonii*-produced L-Lactate, can paradoxically exacerbate vulnerabilities, such as susceptibility to antibiotics. Moreover, our data highlight the context-dependent nature of microbial interactions, emphasizing that a seemingly potent antimicrobial, such as H_2_O_2_, can have both synergistic and antagonistic effects on a microbial community dependent on the environment.

## INTRODUCTION

While microbial pathogenesis studies have often focused on infection with a single organism, it is clear that many bacterial infections do not simply result from colonization by a single microbe, but instead result from colonization by several (1–8). Microbes within polymicrobial infections often display synergy (9–19), defined as an interaction of two or more microbes in an infection site that results in enhanced disease compared to infections containing the individual microbe acting alone (7). The term “synergy” was initially applied to microbiological systems in 1924 by Kämmerer (20) and has subsequently been observed between bacterial species as well as between bacteria, fungi, and viruses (1–8).

Synergy between microbes is often a direct result of the interplay between their metabolic activities and their spatial arrangements relative to each other. However, many microbial communities exist in highly dynamic environments, where these synergistic interactions must be maintained in the face of environmental shifts and the potential for invasion by other microbes. For instance, changes in the pH, oxygen levels, or nutrient levels are common in microbial systems, particularly on the micron scale. These shifts can impact the fitness of synergistic partners or enhance the fitness of new, or low-abundance microbes, facilitating their invasion of the microbial community. Further, human-associated microbes must resist additional challenges in the host environment such as the immune system and antibiotic exposure.

To study the molecular mechanisms controlling synergy and its ecological impacts, we used a two-species model system composed of the oral Gram-negative bacterium *Aggregatibacter actinomycetemcomitans* and the oral Gram-positive bacterium *Streptococcus gordonii*. *A. actinomycetemcomitans* is a non-motile, capnophilic, facultative anaerobe found primarily in the mammalian sub-gingival crevice and on buccal epithelial cells (21, 22). This bacterium is proposed to be a key contributor to the development of localized aggressive periodontitis (23, 24) and implicated in other diseases including endocarditis (25, 26), septicemia (27), and abscesses inside and outside the oral cavity (25). *S. gordonii* is a member of the mitis group of the oral streptococci. The mitis-group streptococci are often the most numerous microbes in the human oral cavity and colonize epithelial cell surfaces and both the sub- and supra-gingival plaque (28). Unlike the mutans-group streptococci, the mitis-group streptococci do not normally cause tooth decay but are opportunistic pathogens that have been isolated from endocardial and septic infections (29).

**Fig 1:**
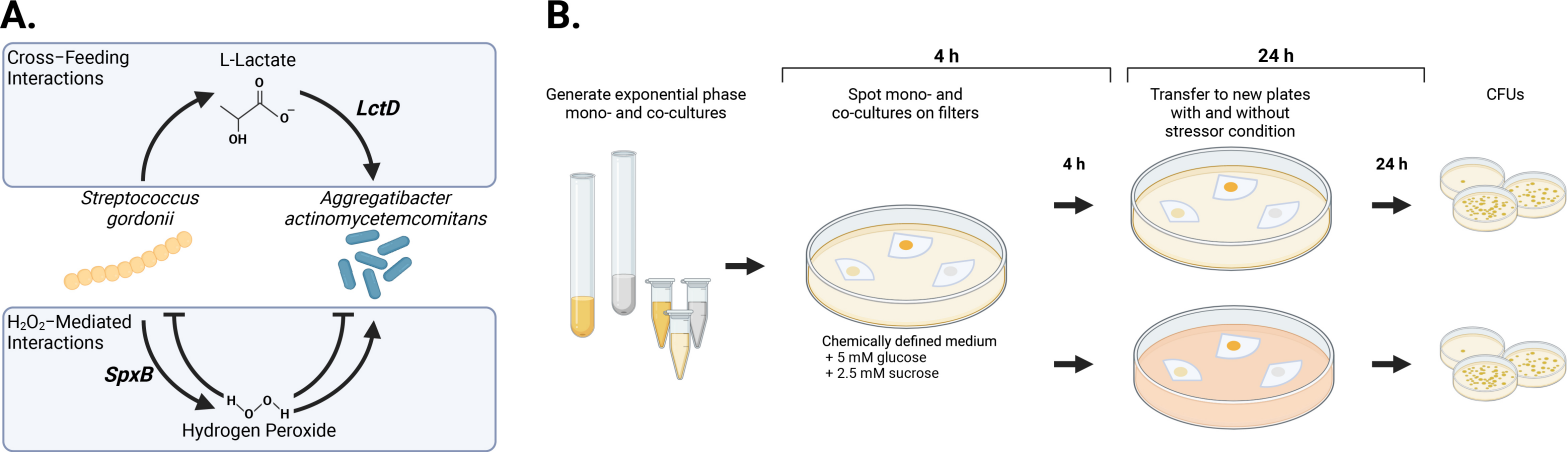
(**A**) Known *in vitro* and *in vivo* interactions between *A. actinomycetemcomitans* and *S. gordonii*. Arrows from L-Lactate and H_2_O_2_ indicate synergistic interactions, and flathead arrows indicate antagonistic interactions. (**B**) Experimental design used to establish colony biofilms and expose them to environmental disturbances. Created with BioRender.com.

We previously discovered that *A. actinomycetemcomitans* and *S. gordonii* display synergy both *in vivo* and *in vitro*, and dependent on the environment, this synergy is controlled by the secretion of L-lactate and H_2_O_2_ by *S. gordonii* (8, 16–19, 30) (Fig. 1A). L-lactate is the preferred carbon source for *A. actinomycetemcomitans* (31, 32), and cross-feeding on *S. gordonii*-produced L-lactate enhances *A. actinomycetemcomitans* fitness in both *in vitro* and *in vivo* co-culture (16, 31–34). This cross-feeding is controlled by PTS substrate exclusion (31, 32), which promotes growth on L-lactate even though *A. actinomycetemcomitans* divides faster and achieves higher cell yields when catabolizing hexose sugars. Dependent on the environment, *S. gordonii* H_2_O_2_ can have positive or negative impacts on *A. actinomycetemcomitans* fitness in co-culture, either serving to directly kill *A. actinomycetemcomitans* at high concentrations (18) or serving as a cue to promote functions critical for co-culture fitness at low concentrations (17, 19).

While the molecular mechanisms controlling synergy in *A. actinomycetemcomitans–S. gordonii* co-infections have been well described, the ecological importance of these interactions have not been widely explored. In this study, we examined how the interactions between *A. actinomycetemcomitans* and *S. gordonii* alter the resilience of this model multi-species community to environmental disturbances. Our results reveal that both L-Lactate and H_2_O_2_ can have either negative or positive impacts on the resilience to stress, dependent on the environment.

## RESULTS AND DISCUSSION

### *A. actinomycetemcomitans* and *S. gordonii* interact via L-lactate and H_2_O_2_ in a colony biofilm model

To study the impact of stressors on *A. actinomycetemcomitans–S. gordonii* co-cultures, we used an *in vitro* colony biofilm model (18, 35) (Fig. 1B). In this model, mono- or co-cultures of *A. actinomycetemcomitans* and *S. gordonii* are spotted onto a 0.2-µm filter on the surface of an agar plate containing chemically defined media (CDM). Glucose (5 mM) and sucrose (2.5 mM) were included in CDM as carbon and energy sources, which promoted growth and co-existence of both microbes. Wild-type (WT) *A. actinomycetemcomitans* and *S. gordonii* were used, along with *A. actinomycetemcomitans ΔlctD*, which does not produce the catabolic L-lactate dehydrogenase and therefore cannot use L-lactate as a carbon and energy source (31). In some cases, *S. gordonii ΔspxB*, which does not produce the enzyme pyruvate oxidase (SpxB) and thus produces significantly less H_2_O_2_ than WT (36), was used to assess the impact of H_2_O_2_ on *S. gordonii* and *A. actinomycetemcomitans* fitness. Bacterial numbers were assessed at an early (4-h) timepoint before significant interactions between *A. actinomycetemcomitans* and *S. gordonii* are expected, as well as a late (28-h) timepoint in which H_2_O_2_- and L-lactate-mediated interactions are prevalent.

As expected, at the 4-h timepoint, there were no differences in colony-forming units (CFUs) for WT *A. actinomycetemcomitans* or *A. actinomycetemcomitans ΔlctD* in mono- and co-culture (Fig. S1), indicating that neither negative or positive interactions were prevalent at this early stage. WT *S. gordonii* also showed no difference in cell numbers at this timepoint whether in mono- or co-culture. These data indicate that both *S. gordonii* and *A. actinomycetemcomitans* strains establish biofilms equivalently at this early stage.

**Fig 2:**
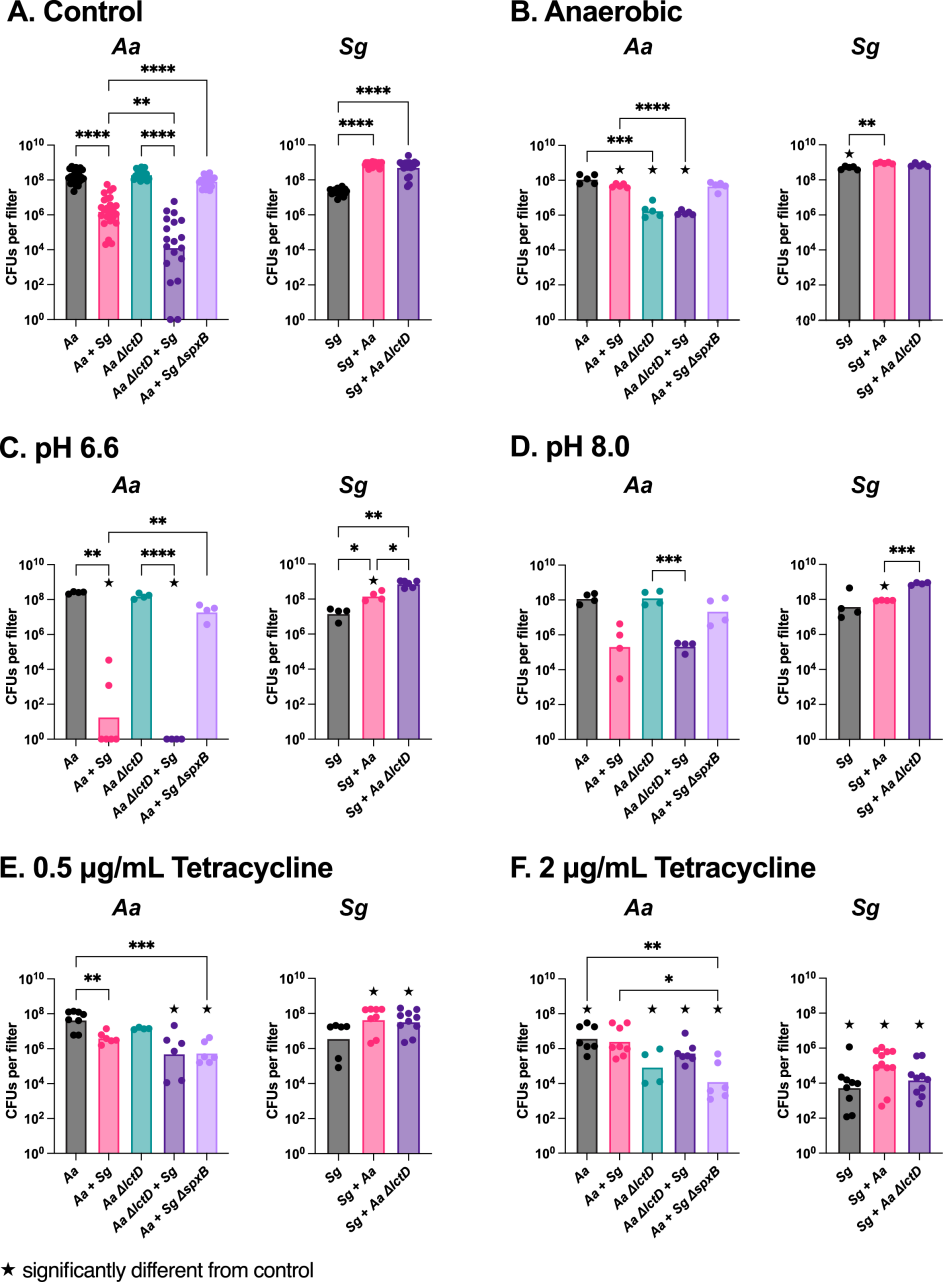
*A. actinomycetemcomitans* (*Aa*) and *S. gordonii* (*Sg*) viable cell numbers in colony biofilms grown with and without environmental stresses. (**A**) *Aa* (left) and *Sg* (right) numbers in colony biofilms on standard CDM without exposure to stress. (**B–F**) *Aa* and *Sg* numbers in colony biofilms after exposure to environmental stresses including (**B**) anaerobic growth on CDM, (**C**) CDM at a pH of 6.6, (**D**) CDM at a pH of 8.0, (**E**) CDM with 0.5 µg/mL of tetracycline, (**F**) CDM with 2.0 µg/mL of tetracycline. Statistical differences were determined by a Dunnett’s T3 multiple comparisons test and are indicated as *P < 0.05; **P < 0.01; ****P < 0.0001. ★ indicates significantly different from the same condition in the control condition in (**A**).

After 4 h, we transferred the colony biofilms to new CDM plates, incubated them for an additional 24 h, and quantified viable bacterial numbers. Co-culture with either WT *A. actinomycetemcomitans* or the *A. actinomycetemcomitans ΔlctD* mutant increased the *S. gordonii* numbers over 10-fold relative to mono-culture (Fig. 2A). This increase was expected as *S. gordonii* produces extremely high levels of H_2_O_2_ *in vitro*, which can be toxic to this bacterium despite its relatively high resistance to this antimicrobial (37–39). However, *A. actinomycetemcomitans* has been shown to detoxify H_2_O_2_ during co-culture via the cytoplasmic enzyme KatA (encoding catalase), which enhances *S. gordonii* fitness during co-infection (17, 18, 40). The role of H_2_O_2_ in mediating enhanced *S. gordonii* fitness in co-culture was further supported by the observation that eliminating the ability of *S. gordonii* to produce H_2_O_2_ via SpxB (*S. gordonii ΔspxB*) enhanced *S. gordonii* growth yields in the colony biofilm model by over 10-fold at the 28-h timepoint (Fig. S2).

In contrast, WT *A. actinomycetemcomitans* exhibited an ~100-fold decrease in bacterial numbers in co-culture with *S. gordonii* relative to mono-culture at the 28-h timepoint (Fig. 2A). Based on our previous results (17, 18), we hypothesized that this decrease was a result of high levels of H_2_O_2_ production by *S. gordonii*. To test this, WT *A. actinomycetemcomitans* was co-cultured with *S. gordonii ΔspxB*, and bacterial numbers were quantified after 28 h. *A. actinomycetemcomitans* numbers in co-culture with the *ΔspxB* mutant were equivalent to those of the mono-culture (Fig. 2A), while *S. gordonii ΔspxB* numbers were similar to those of WT *S. gordonii* in co-culture (Fig. S2). These data indicate that *S. gordonii* production of H_2_O_2_ primarily mediates the decrease in WT *A. actinomycetemcomitans* during co-culture with WT *S. gordonii* in the colony biofilm model. Notably, the *A. actinomycetemcomitans ΔlctD* mutant, which cannot catabolize L-lactate, exhibited an ~1,000-fold decrease in growth yield during co-culture with WT *S. gordonii* (Fig. 2A). This decrease was significantly lower than WT *A. actinomycetemcomitans* during co-culture. Thus, while *A. actinomycetemcomitans* levels decrease in the colony biofilm model during co-culture with *S. gordonii*, the ability to cross-feed on *S. gordonii*-produced L-lactate still imparts a significant increase in *A. actinomycetemcomitans* fitness (~10-fold in bacterial numbers) during co-culture.

### Impact of stressors on *A. actinomycetemcomitans* and *S. gordonii* fitness in mono- and co-culture

**Fig 3:**
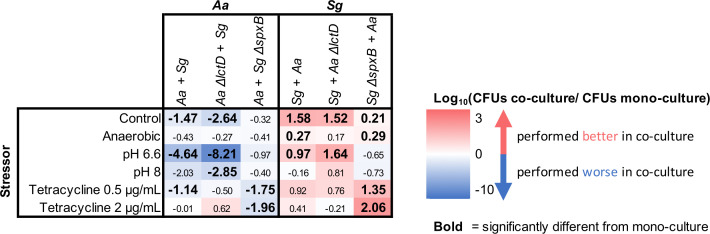
Summary of results from environmental disturbance experiments in Fig. 2. Heatmap showing the impact of co-culture on *A. actinomycetemcomitans* (*Aa*) and *S. gordonii* (*Sg*) numbers with and without disturbances. Numbers in the heatmap are the log_10_-transformed ratio of CFUs in co-culture:CFUs in mono-culture. Positive numbers indicate a bacterium did better in co-culture and a negative number indicates a bacterium did worse in co-culture. Bold numbers are statistically significant changes relative to mono-culture (*P* < 0.05). Statistical differences were determined by a Dunnett’s T3 multiple comparisons test.

To study the impact of environmental stressors on *A. actinomycetemcomitans* and *S. gordonii* fitness during co-culture, membranes containing 4-h biofilms were moved to CDM agar plates containing stressors and cultured for an additional 24 h. Five stressor conditions were used as follows: anaerobic growth, acidic pH (pH = 6.6), alkaline pH (pH = 8.0), tetracycline at 0.5 µg/mL, and tetracycline at 2 µg/mL. Results from each stress condition were compared to those of the no stress condition (CDM, Fig. 2A). Specific findings are discussed below and summarized in Fig. 3.

#### Anaerobic growth

Members of the oral microbiome encounter both aerobic and anaerobic conditions in the oral cavity, and oxygen levels can impact the outcome of microbial interactions, including those between *A. actinomycetemcomitans* and *S. gordonii* (16–19). For example, both the LctD and SpxB enzymes require O_2_. Thus, *A. actinomycetemcomitans* does not catabolize L-lactate, and *S. gordonii* does not produce H_2_O_2_ under anaerobic conditions (17, 36). *A. actinomycetemcomitans* showed a significant increase (~50-fold) in bacterial numbers following anaerobic co-culture compared to aerobic co-culture growth (Fig. 2B), with WT *A. actinomycetemcomitans* having similar numbers following mono- and co-culture anaerobic growth. These results further support that H_2_O_2_ production by *S. gordonii* is the primary mechanism of *A. actinomycetemcomitans* inhibition under our aerobic conditions. A similar increase in the numbers of *A. actinomycetemcomitans ΔlctD* (~100-fold) was also observed in anaerobic co-culture, again likely due to the lack of H_2_O_2_ production by *S. gordonii* under these conditions. Unexpectedly, *A. actinomycetemcomitans ΔlctD* showed an ~100-fold decrease in bacterial numbers after mono-culture anaerobic growth compared to aerobic growth, similar levels to those observed for this mutant in anaerobic co-culture. The mechanism of the anaerobic growth yield defect of *A. actinomycetemcomitans ΔlctD* on CDM with glucose is not known, as LctD is a catabolic L-lactate dehydrogenase (31, 32) that does not impact anaerobic catabolism of glucose (16). Finally, *S. gordonii* increased in mono-culture during anaerobic growth, further supporting that H_2_O_2_ is inhibiting *S. gordonii* mono-culture growth aerobically. Together, these data reveal that anaerobic growth impacts the fitness of both *A. actinomycetemcomitans* and *S. gordonii* primarily through the lack of H_2_O_2_ production by *S. gordonii* in the absence of oxygen, and L-lactate cross-feeding has no impact on fitness. Although it was less notable than in the control, *S. gordonii* continued to show higher fitness in co-culture compared to mono-culture under anaerobic conditions. We suspect that this was likely due to interactions between *A. actinomycetemcomitans* and *S. gordonii* during the 4-h aerobic establishment-phase incubation. However, it is also possible that *A. actinomycetemcomitans* confers additional, non-oxygen-dependent growth benefits to *S. gordonii*.

#### Acidic and alkaline pH

pH changes are common in most environments including the oral cavity where microbes must combat exposure to both acidic and alkaline pH. To examine how changes in pH impacts the fitness of *A. actinomycetemcomitans* and *S. gordonii*, we exposed 4-h biofilms to CDM at pH 6.6 (Fig. 2C) and 8.0 (Fig. 2D). *A. actinomycetemcomitans* numbers were significantly impacted during co-culture growth at a pH of 6.6 compared to those of mono-culture growth, reducing growth yields >10^7^-fold. This reduction in *A. actinomycetemcomitans* numbers required production of H_2_O_2_ by *S. gordonii* as co-culture with the *ΔspxB* mutant increased *A. actinomycetemcomitans* growth yields to mono-culture levels (Fig. 2C). A statistically significant decrease in *S. gordonii* numbers during co-culture with WT *A. actinomycetemcomitans* was observed at a pH of 6.6 (Fig. 2C), and this decrease was also observed at a pH of 8.0 (Fig. 2D). However, co-culture with *A. actinomycetemcomitans ΔlctD* did not result in this decrease in *S. gordonii* numbers (Fig. 2C and D). Collectively, these results indicate that (i) co-culture significantly decreases the fitness of *A. actinomycetemcomitans* at low pH, and this is dependent on H_2_O_2_ production by *S. gordonii*; (ii) *S. gordonii* co-culture numbers decrease upon exposure to both acidic and alkaline pH, and the ability of *A. actinomycetemcomitans* to consume L-lactate is critical to reduce *S. gordonii* fitness in co-culture at a non-neutral pH. While our genetic studies provide strong evidence for a role of H_2_O_2_ and L-lactate, further studies are required to fully elucidate the mechanisms responsible for these observations.

#### Tetracycline exposure

Interactions between bacteria, including cross-feeding, can alter susceptibility to antimicrobial killing (10, 41–43). Tetracycline has been used in oral care for decades, including via localized delivery to help treat periodontal disease (44–46). To assess how bacterial interactions impact susceptibility to this antimicrobial, 4-h colony biofilms were exposed for 24 h to levels of tetracycline below (0.5 µg/mL) and at (2 µg/mL) the minimum inhibitory concentrations (MIC). WT *A. actinomycetemcomitans* showed little response in mono- and co-culture to sub-MIC levels of tetracycline, except when co-cultured with *S. gordonii ΔspxB* where growth yields decreased ~100-fold compared to the control (Fig. 2E). This was also observed at the higher level of tetracycline in which *A. actinomycetemcomitans* co-culture with *S. gordonii ΔspxB* showed an approximate 100-fold decrease in growth yields compared to the control and compared to co-culture with WT *S. gordonii* (Fig. 2F). These data indicate that H_2_O_2_ production by *S. gordonii* enhances *A. actinomycetemcomitans* tolerance to tetracycline. *A. actinomycetemcomitans ΔlctD* showed a significant increase in growth yields during co-culture with *S. gordonii* in the presence of sub-MIC tetracycline compared to the control (Fig. 2E), and this was also observed at the higher level of tetracycline (Fig. 2F). *S. gordonii* displayed decreases in growth yields in most cases, particularly at higher tetracycline levels.

Together, these data indicate that interactions between *A. actinomycetemcomitans* and *S. gordonii* are critical for *A. actinomycetemcomitans* tetracycline tolerance. While the mechanism(s) for the altered tolerances to tetracycline are not known, this is likely not due to differential regulation of genes known to be critical for antimicrobial tolerance. Indeed, transcriptome studies of *A. actinomycetemcomitans* upon exposure to H_2_O_2_ and during growth on L-lactate did not show differential expression of efflux pumps or other genes known to be involved in tetracycline tolerance (17, 31).

### The impact of *S. gordonii* and *A. actinomycetemcomitans* mono- and co-culture on microbial invasion *in vitro* and *in vivo*

**Fig 4:**
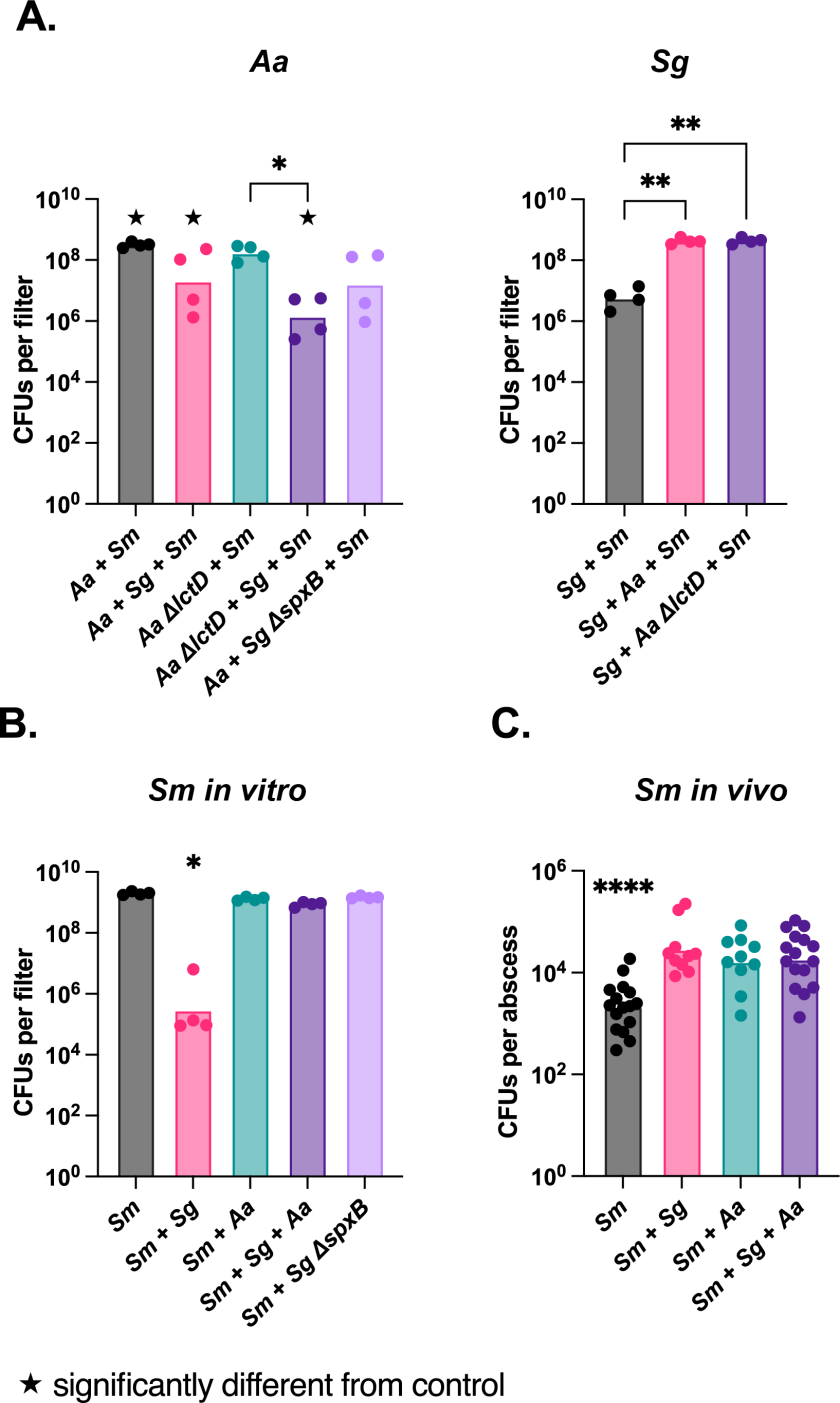
Invasion of *A. actinomycetemcomitans* (*Aa*) and *S. gordonii* (*Sg*) communities by *S. marcescens (Sm*) *in vitro* and *in vivo*. (**A**) *Aa* (left) and *Sg* (right) numbers during *in vitro* growth in the colony biofilm with *Sm*. (**B**) *Sm* numbers in the *in vitro* colony biofilm model with *Aa* and/or *Sg*. (**C**) *Sm* numbers in the murine abscess model with *Aa* and/or *Sg*. Co-culture with *Aa* or *Sg* increases *Sm* growth and survival in a mouse-infection model. Statistical differences were determined by a Dunnett’s T3 multiple comparisons test and are indicated as *P < 0.05; **P < 0.01; ****P < 0.0001. ★ indicates significantly different from the same condition in the control condition from Fig. 2A.

In addition to the five environmental stressors, we also assessed how *A. actinomycetemcomitans* and *S. gordonii* communities responded to invasion of the community by a third bacterium. For these experiments, we used the Gram-negative bacterium *Serratia marcescens* as the “invader.” This bacterium was chosen as we previously showed that among 25 microbes, it achieved the highest numbers when competed against *A. actinomycetemcomitans* in an *in vivo* murine abscess model (47). For these experiments, colony biofilms were created as outlined above, except *S. marcescens* was included in the biofilm inoculum at 100–1,000 times fewer bacteria than *A. actinomycetemcomitans* and/or *S. gordonii*. These three species biofilms were incubated for 24 h before quantifying bacterial number by CFUs. While *S. gordonii* grew to similar levels with or without *S. marcescens*, *A. actinomycetemcomitans* showed slight increases in growth yields in the three species biofilm compared to co-culture with *S. gordonii* (Fig. 4A). *S. marcescens* grew to ~10^9^ CFUs/biofilm in all conditions except when co-cultured with WT *S. gordonii*, where levels reached ~10^5^ CFU/biofilm (Fig. 4B). This was due to H_2_O_2_ production by *S. gordonii*, as co-culture of *S. marcescens* with *S. gordonii ΔspxB* reached ~10^9^ CFU/biofilm (Fig. 4B). These data indicate that H_2_O_2_ production significantly impacts the ability of *S. marcescens* to invade *S. gordonii* biofilms, but introduction of *A. actinomycetemcomitans* into this biofilm allows *S. marcescens* to invade, likely through detoxification of H_2_O_2_ as previously described (18, 48).

As we identified *S. marcescens* as a potentially potent bacterial invader using data generated in a murine abscess model (47), we next assessed invasion by *S. marcescens* into an *A. actinomycetemcomitans* and/or *S. gordonii* community using this model. In this model, bacteria are inoculated in the inner thigh of the mouse, which generates an abscess within 3 days that can easily be removed to assess bacterial numbers. Abscesses were infected with *S. marcescens* mono-cultures, *S. marcescens* in co-culture with *A. actinomycetemcomitans* or *S. gordonii*, or all three species together. *S. marcescens* was inoculated at 100 times lower levels than the other bacteria, while maintaining the same overall inoculum in all experiments. After 3 days, abscesses were removed and bacterial numbers enumerated. In mono-infection, *S. marcescens* was recovered at 2.2 × 10^3^ CFUs/abscess. However, *S. marcescens* numbers increased significantly (between 10- and 50-fold) in all co-infection conditions (Fig. 4C). *A. actinomycetemcomitans* numbers were the same in mono- and co-culture abscesses, and *S. gordonii* numbers were the same in the mono- and co-culture with *S. marcescens* abscesses but decreased in three species abscesses (Fig. S3). Thus, in the murine abscess, the presence of *A. actinomycetemcomitans* and/or *S. gordonii* increased abundance of *S. marcescens*, but interactions between *A. actinomycetemcomitans* and *S. gordonii* did not alter *S. marcescens* abundance.

### Conclusions and future directions

Pairwise studies of *A. actinomycetemcomitans* and *S. gordonii*, both in *in vitro* and *in vivo*, have provided significant new insights into the synergistic and antagonistic interactions that govern microbial community biogeography and function. Here, we investigated how interactions between these microbes impact their fitness when confronted with environmental changes, focusing on the *S. gordonii*-produced extracellular molecules L-lactate and H_2_O_2_. Our results revealed that while the ability of *A. actinomycetemcomitans* to catabolize L-lactate enhanced its fitness during co-culture with *S. gordonii*, this function had little impact on survival to most environmental changes. The most intriguing finding was that *A. actinomycetemcomitans ΔlctD* showed a significant increase in growth yields during co-culture with *S. gordonii* in the presence of tetracycline, indicating that the ability to degrade L-lactate might be detrimental to *A. actinomycetemcomitans* in co-culture in the presence of this antimicrobial. H_2_O_2_ largely had negative impacts in this colony biofilm model for both microbes, and this is not particularly surprising given the high levels of H_2_O_2_ produced by *S. gordonii in vitro* (37–39). However, our results provided new insights into the positive effect of H_2_O_2_ on *A. actinomycetemcomitans*, with H_2_O_2_ production by *S. gordonii* enhancing *A. actinomycetemcomitans* tolerance to tetracycline (Fig. 2E and F). Future work will be focused on determining the mechanism controlling this enhanced tolerance, which we propose will likely occur by H_2_O_2_-mediated transcriptional induction of functions critical for tetracycline tolerance. Finally, we observed minimal impact of co-culture on the ability of *S. marcescens* to invade our model communities both *in vitro* and *in vivo*. However, the fact that *S. gordonii* inhibited *S. marcescens in vitro* but enabled invasion *in vivo* reinforces the idea of the importance of the environment for understanding the relevance of microbial interactions.

## MATERIALS AND METHODS

### Strains, media, and growth conditions

*A. actinomycetemcomitans* strains *VT1169* (49) and *VT1169 ΔlctD* (31) and *S. gordonii* strains *Challis DL1.1* (ATCC 49818) and *Challis DL1.1 ΔspxB* (36) were used. *S. marcescens Db11* (50) was used in the invasion condition. Liquid cultures were grown in filter-sterilized tryptic soy broth supplemented with 0.5% yeast extract (TSBYE) and incubated at 37°C in a 5% CO_2_ atmosphere. *A. actinomycetemcomitans* was grown with shaking at 220 rpm overnight, then back-diluted 1:5 into TSBYE. *S. gordonii* strains were incubated statically overnight, then back-diluted 1:10 into TSBYE. *S. marcescens* strains were grown with shaking at 220 rpm overnight, then back-diluted 1:20 in TSBYE. All cultures were then incubated for an additional 2 h to ensure the cells were in the exponential phase. For the comparison of mono-culture and co-culture growth, agar plates of a modified Socransky’s CDM for oral microbes (51) containing 5 mM D-glucose and 2.5 mM D-sucrose were used. The CDM recipe used included 1 mg/L of menadione, 5 mg/L of hemin, and omitted putrescine dihydrochloride or DL-mevalonic acid lactone. For CFU counts and selection plating, tryptic soy agar supplemented with 0.5% yeast extract (TSAYE) was used. Where applicable, TSAYE was supplemented with antibiotics. In non-invasion conditions, 5 µg/mL of vancomycin was used to select for *A. actinomycetemcomitans*, 50 µg/mL of streptomycin was used to select for WT *S. gordonii*, and 50 µg/mL of spectinomycin was used to select for *S. gordonii ΔspxB*. In invasion conditions, 5 µg/mL of vancomycin and room-temperature incubation were used to select for *S. marcescens*, rifampin 150 µg/mL was used to select for *A. actinomycetemcomitans*, 50 µg/mL of streptomycin plus 2.46 mL/L of phenylethyl alcohol (PEA) was used to select for WT *S. gordonii*, and 50 µg/mL of spectinomycin plus 2.46 mL/L of PEA were used to select for *S. gordonii ΔspxB*. Under aerobic growth conditions, CDM plates were stored at 37°C in a 5% CO_2_ incubator. Under anaerobic growth conditions, the plates were incubated at 37°C in an anaerobic chamber with a 5% H_2_, 10% CO_2_, and 85% N_2_ atmosphere.

### Preparation of mono- and co-cultures for environmental stress experiments

All four *A. actinomycetemcomitans* and *S. gordonii* strains were grown in liquid culture as previously described. One milliliter of each culture was spun down at 9.2 × *g* in a Spectrafuge 24D Digital Lab Microcentrifuge and washed twice with sterile phosphate-buffered saline (PBS, pH = 7.4) to remove residual nutrients from the TSBYE. *A. actinomycetemcomitans* strains were diluted in PBS to an OD_600_ of 0.08 (8 × 10^7^ CFUs/mL), and *S. gordonii* strains were diluted in PBS to an OD_600_ of 0.008 (8 × 10^6^ CFUs/mL). Mono-cultures were prepared by combining 250 µL of culture with 250 µL of PBS. Co-cultures were prepared by combining 250 µL of two different strains.

Nuclepore Track-Etch Membrane 0.2-µm filters were placed on CDM plates using sterile tweezers with the hydrophobic side facing up. Each filter was inoculated with 25 µL of the appropriate culture to achieve an approximate inoculum of 1 × 10^6^ CFUs for *A. actinomycetemcomitans* strains and 1 × 10^5^ CFUs for *S. gordonii* strains. Plates were placed in the biosafety cabinet until the droplets dried, then the plates were incubated at 37°C in a 5% CO_2_ atmosphere. After 4 h, each filter was transferred to fresh CDM plates with and without a stress condition. The plates were incubated for an additional 24 h. At the 4- and 28-h timepoints, filters were removed with ethanol and flame-sterilized tweezers and placed into individual 2.8-mm stainless steel-beaded BeadBugTM homogenizer tubes containing 1 mL of PBS. The tubes were placed in a Mini-Beadbeater-16 (BioSpec Products) for 30 s. CFU of the resulting samples were obtained via spread-plated serial dilutions. Mono-cultures were plated on TSAYE without antibiotics. Co-cultures were plated on two TSAYE plates containing antibiotics, one that selected for *S. gordonii* and the other that selected for *A. actinomycetemcomitans*. All plates were incubated at 37°C in a 5% CO_2_ atmosphere until countable.

### Effect of anaerobic conditions on mono- and co-cultures

Mono- and co-cultures were grown aerobically on CDM for 4 h as described above. The filters were transferred sterilely to both oxic and anoxic CDM plates. The anoxic CDM plates were incubated anaerobically, and the oxic CDM plates were incubated aerobically as a control.

### Effect of pH on mono- and co-cultures

While preparing the media, we measured the pH of our base CDM with a pH electrode prior to sterilization or the addition of agar. We adjusted the pH to the desired values via dropwise additions of 1 M HCl or 1 M NaOH. Mono- and co-cultures were grown on pH 7.2 CDM for 4 h as described above. The filters were sterilely transferred to CDM plates with a pH of either 6.6 or 8.0, and CDM plates with a pH of 7.2 were used as the control. Immediately after biofilm collection, we used both a liquid pH indicator (Universal pH indicator solution, Spectrum Chemical) and litmus paper to confirm that the plates maintained their intended pH values throughout the experiment.

### Effect of tetracycline on mono-cultures and co-cultures

Mono- and co-cultures were prepared and incubated on CDM containing no antibiotics for 4 h as described above. The filters were transferred sterilely to CDM plates containing tetracycline at either 0.5 or 2 µg/mL. Additional filters were transferred to CDM plates containing no tetracycline as a control. After 24 h, the filters were sterilely transferred to unused CDM plates containing no antibiotics and allowed to sit at room temperature for 30 min to allow residual tetracycline to disperse out of the samples before quantifying bacterial numbers using CFUs.

### *S. marcescens in vitro* invasion experiments

All five bacterial strains were grown in liquid culture as described above. One milliliter of each culture was spun down at 9.2 × *g* in a Spectrafuge 24D Digital Lab Microcentrifuge and washed twice with sterile PBS to remove residual nutrients from the TSBYE. *A. actinomycetemcomitans* strains were diluted in PBS to an OD_600_ of 0.12 (1.2 × 10^8^ CFUs/mL), *S. gordonii* strains were diluted in PBS to an OD_600_ of 0.012 (1.2 × 10^7^ CFUs/mL), and *S. marcescens* was diluted in PBS to an OD_600_ of 0.00012 (1.2 × 10^5^ CFUs/mL). Mono-cultures were prepared by combining 250 µL of culture with 500 µL of PBS. Co-cultures were prepared by combining 250 µL of two different strains and 250 µL of PBS. Three species cultures were prepared by combining 250 µL of three different strains. Of the appropriate culture, 25 µL was inoculated onto each filter as described above. Mono-cultures were plated on TSAYE without antibiotics. Co-cultures and three-species biofilms were plated on multiple TSAYE plates containing antibiotics to select for each of the strain present. *S. marcescens* mono-culture and *S. marcescens*-selective plates were incubated at room temperature until countable. The remaining plates were incubated at 37°C in a 5% CO_2_ until countable.

### *S. marcescens in vivo* invasion experiments

The murine abscess model was performed as previously described (47). Briefly, all bacterial strains were grown in liquid culture as described above. Each culture was spun down at 9.2 × *g* in a Spectrafuge 24D Digital Lab Microcentrifuge and washed once with sterile PBS to remove residual nutrients from the TSBYE. *A. actinomycetemcomitans* and *S. gordonii* strains were diluted in sterile saline to an OD_600_ of 4 (4 × 10^9^ CFUs/mL); *S. marcescens* was diluted in sterile saline to an OD_600_ of 0.04 (4 × 10^7^ CFUs/mL). Inocula were prepared by diluting cultures into sterile saline to a final volume of 1 mL: as appropriate, the total volume of *A. actinomycetemcomitans* plus *S. gordonii* was maintained as 20% of the final inoculum (200 µL), and the total volume of *S. marcescens* was maintained as 10% of the final inoculum (100 µL). Ten-week-old male and female Swiss Webster mice were purchased from Charles River and acclimated for at least 3 days before experimentation. Abscesses were initiated by injecting 100 µL of each mixture into the inner thigh to aim for an initial inoculum of 1 × 10^7^ for *A. actinomycetemcomitans* and *S. gordonii* and 1 × 10^5^ for *S. marcescens*. Abscesses (10 to 16) were performed per condition, powered to detect less than a 1-log difference between conditions (47). Animals were given *ad libitum* access to food and water. Animals were euthanized, and abscesses were harvested after 3 days. Abscesses were homogenized in 900 µL of PBS for 30 s in BeadBug tubes (Sigma-Aldrich) with 2.8-mm steel beads using a Mini-Beadbeater-16 (BioSpec Products) and the homogenate plated onto selective plates as described above to quantify numbers of each bacterium. This protocol was approved by the Institutional Animal Care and Use Committees at Georgia Institute of Technology (protocol no. A100127E).

### Statistical analyses

Statistical differences throughout the paper were determined in GraphPad Prism by Dunnett’s T3 multiple comparisons tests. The first set of tests compared the log CFU values within a single bacterial species and environmental disturbance. Then, in a separate Dunnett’s T3 multiple comparisons test, the log CFUs from the control condition were compared with the corresponding log CFUs from the disturbance conditions. No outliers were removed in our analyses.

## SUPPLEMENTAL MATERIAL

Supplemental material spectrum.01005-24-s0001.docx Fig. S1 to S3.

ASM does not own the copyrights to Supplemental Material that may be linked to, or accessed through, an article. The authors have granted ASM a non-exclusive, world-wide license to publish the Supplemental Material files. Please contact the corresponding author directly for reuse.

## References

[1] Anju VT, Busi S, Imchen M, Kumavath R, Mohan MS, Salim SA, Subhaswaraj P, Dyavaiah M. 2022. Polymicrobial infections and biofilms: clinical significance and eradication strategies. Antibiotics (Basel) 11:1731. doi:10.3390/antibiotics11121731 36551388 PMC9774821

[2] Azimi S, Lewin GR, Whiteley M. 2022. The biogeography of infection revisited. Nat Rev Microbiol 20:579–592. doi:10.1038/s41579-022-00683-3 35136217 PMC9357866

[3] Bakaletz LO. 2004. Developing animal models for polymicrobial diseases. Nat Rev Microbiol 2:552–568. doi:10.1038/nrmicro928 15197391 PMC7097426

[4] Brouqui P, Raoult D. 2001. Endocarditis due to rare and fastidious bacteria. Clin Microbiol Rev 14:177–207. doi:10.1128/CMR.14.1.177-207.2001 11148009 PMC88969

[5] Jenkinson HF, Lamont RJ. 2005. Oral microbial communities in sickness and in health. Trends Microbiol 13:589–595. doi:10.1016/j.tim.2005.09.006 16214341

[6] Kuramitsu HK, He X, Lux R, Anderson MH, Shi W. 2007. Interspecies interactions within oral microbial communities. Microbiol Mol Biol Rev 71:653–670. doi:10.1128/MMBR.00024-07 18063722 PMC2168648

[7] Murray JL, Connell JL, Stacy A, Turner KH, Whiteley M. 2014. Mechanisms of synergy in polymicrobial infections. J Microbiol 52:188–199. doi:10.1007/s12275-014-4067-3 24585050 PMC7090983

[8] Stacy A, McNally L, Darch SE, Brown SP, Whiteley M. 2016. The biogeography of polymicrobial infection. Nat Rev Microbiol 14:93–105. doi:10.1038/nrmicro.2015.8 26714431 PMC5116812

[9] Chen PB, Davern LB, Katz J, Eldridge JH, Michalek SM. 1996. Host responses induced by co-infection with Porphyromonas gingivalis and Actinobacillus actinomycetemcomitans in a murine model. Oral Microbiol Immunol 11:274–281. doi:10.1111/j.1399-302x.1996.tb00181.x 9002881

[10] Ibberson CB, Barraza JP, Holmes AL, Cao P, Whiteley M. 2022. Precise spatial structure impacts antimicrobial susceptibility of S. aureus in polymicrobial wound infections. Proc Natl Acad Sci U S A 119:e2212340119. doi:10.1073/pnas.2212340119 36520668 PMC9907066

[11] Ibberson CB, Stacy A, Fleming D, Dees JL, Rumbaugh K, Gilmore MS, Whiteley M. 2017. Co-infecting microorganisms dramatically alter pathogen gene essentiality during polymicrobial infection. Nat Microbiol 2:17079. doi:10.1038/nmicrobiol.2017.79 28555625 PMC5774221

[12] Kesavalu L, Holt SC, Ebersole JL. 1998. Virulence of a polymicrobic complex, Treponema denticola and Porphyromonas gingivalis, in a murine model. Oral Microbiol Immunol 13:373–377. doi:10.1111/j.1399-302x.1998.tb00694.x 9872114

[13] Kozarov EV, Dorn BR, Shelburne CE, Dunn Jr WA, Progulske-Fox A. 2005. Human atherosclerotic plaque contains viable invasive Actinobacillus actinomycetemcomitans and Porphyromonas gingivalis. Arterioscler Thromb Vasc Biol 25:e17–e18. doi:10.1161/01.ATV.0000155018.67835.1a 15662025

[14] Mastropaolo MD, Evans NP, Byrnes MK, Stevens AM, Robertson JL, Melville SB. 2005. Synergy in polymicrobial infections in a mouse model of type 2 diabetes. Infect Immun 73:6055–6063. doi:10.1128/IAI.73.9.6055-6063.2005 16113326 PMC1231087

[15] Nagashima H, Takao A, Maeda N. 1999. Abscess forming ability of Streptococcus milleri group: synergistic effect with Fusobacterium nucleatum. Microbiol Immunol 43:207–216. doi:10.1111/j.1348-0421.1999.tb02395.x 10338189

[16] Ramsey MM, Rumbaugh KP, Whiteley M. 2011. Metabolite cross-feeding enhances virulence in a model polymicrobial infection. PLoS Pathog 7:e1002012. doi:10.1371/journal.ppat.1002012 21483753 PMC3069116

[17] Ramsey MM, Whiteley M. 2009. Polymicrobial interactions stimulate resistance to host innate immunity through metabolite perception. Proc Natl Acad Sci U S A 106:1578–1583. doi:10.1073/pnas.0809533106 19164580 PMC2629492

[18] Stacy A, Everett J, Jorth P, Trivedi U, Rumbaugh KP, Whiteley M. 2014. Bacterial fight-and-flight responses enhance virulence in a polymicrobial infection. Proc Natl Acad Sci U S A 111:7819–7824. doi:10.1073/pnas.1400586111 24825893 PMC4040543

[19] Stacy A, Fleming D, Lamont RJ, Rumbaugh KP, Whiteley M. 2016. A commensal bacterium promotes virulence of an opportunistic pathogen via cross-respiration. mBio 7:e00782-16. doi:10.1128/mBio.00782-16 27353758 PMC4916382

[20] Kämmerer H. 1924. Beiträge zur bedeutung des bakteriellen synergismus für die biologie. Klin Wochenschr 3:723–727. doi:10.1007/BF01737194

[21] Kroes I, Lepp PW, Relman DA. 1999. Bacterial diversity within the human subgingival crevice. Proc Natl Acad Sci U S A 96:14547–14552. doi:10.1073/pnas.96.25.14547 10588742 PMC24473

[22] Paster BJ, Boches SK, Galvin JL, Ericson RE, Lau CN, Levanos VA, Sahasrabudhe A, Dewhirst FE. 2001. Bacterial diversity in human subgingival plaque. J Bacteriol 183:3770–3783. doi:10.1128/JB.183.12.3770-3783.2001 11371542 PMC95255

[23] Meyer DH, Fives-Taylor PM. 1998. Oral pathogens: from dental plaque to cardiac disease. Curr Opin Microbiol 1:88–95. doi:10.1016/s1369-5274(98)80147-1 10066462

[24] Slots J, Reynolds HS, Genco RJ. 1980. Actinobacillus actinomycetemcomitans in human periodontal disease: a cross-sectional microbiological investigation. Infect Immun 29:1013–1020. doi:10.1128/iai.29.3.1013-1020.1980 6968718 PMC551232

[25] Kaplan AH, Weber DJ, Oddone EZ, Perfect JR. 1989. Infection due to Actinobacillus actinomycetemcomitans: 15 cases and review. Rev Infect Dis 11:46–63. doi:10.1093/clinids/11.1.46 2644690

[26] Paturel L, Casalta JP, Habib G, Nezri M, Raoult D. 2004. Actinobacillus actinomycetemcomitans endocarditis. Clin Microbiol Infect 10:98–118. doi:10.1111/j.1469-0691.2004.00794.x 14759235

[27] Shalini S, Ganesh P, Anand AR. 1995. Actinobacillus actinomycetemcomitans septicemia during pregnancy. Int J Gynaecol Obstet 51:57–58. doi:10.1016/0020-7292(95)80010-a 8582520

[28] Okahashi N, Nakata M, Kuwata H, Kawabata S. 2022. Oral mitis group streptococci: a silent majority in our oral cavity. Microbiol Immunol 66:539–551. doi:10.1111/1348-0421.13028 36114681

[29] Chhatwal S. 2007. Molecular biology of streptococci. Horizon Scientific Press, Wymondham, U.K.

[30] Stacy A, Abraham N, Jorth P, Whiteley M. 2016. Microbial community composition impacts pathogen iron availability during polymicrobial infection. PLoS Pathog 12:e1006084. doi:10.1371/journal.ppat.1006084 27973608 PMC5156373

[31] Brown SA, Whiteley M. 2007. A novel exclusion mechanism for carbon resource partitioning in Aggregatibacter actinomycetemcomitans. J Bacteriol 189:6407–6414. doi:10.1128/JB.00554-07 17586632 PMC1951915

[32] Brown SA, Whiteley M. 2009. Characterization of the L-lactate dehydrogenase from Aggregatibacter actinomycetemcomitans. PLoS One 4:e7864. doi:10.1371/journal.pone.0007864 19924225 PMC2773005

[33] Fine DH, Schreiner H, Velusamy SK. 2020. Aggregatibacter, a low abundance pathobiont that influences biogeography, microbial dysbiosis, and host defense capabilities in periodontitis: the history of a bug, and localization of disease. Pathogens 9:179. doi:10.3390/pathogens9030179 32131551 PMC7157720

[34] Velusamy SK, Sampathkumar V, Ramasubbu N, Paster BJ, Fine DH. 2019. Aggregatibacter actinomycetemcomitans colonization and persistence in a primate model. Proc Natl Acad Sci U S A 116:22307–22313. doi:10.1073/pnas.1905238116 31611409 PMC6825321

[35] Koley D, Ramsey MM, Bard AJ, Whiteley M. 2011. Discovery of a biofilm electrocline using real-time 3D metabolite analysis. Proc Natl Acad Sci U S A 108:19996–20001. doi:10.1073/pnas.1117298108 22123963 PMC3250129

[36] Kreth J, Zhang Y, Herzberg MC. 2008. Streptococcal antagonism in oral biofilms: Streptococcus sanguinis and Streptococcus gordonii interference with Streptococcus mutans. J Bacteriol 190:4632–4640. doi:10.1128/JB.00276-08 18441055 PMC2446780

[37] Itzek A, Zheng L, Chen Z, Merritt J, Kreth J. 2011. Hydrogen peroxide-dependent DNA release and transfer of antibiotic resistance genes in Streptococcus gordonii. J Bacteriol 193:6912–6922. doi:10.1128/JB.05791-11 21984796 PMC3232836

[38] Xu Y, Itzek A, Kreth J. 2014. Comparison of genes required for H_2_O_2_ resistance in Streptococcus gordonii and Streptococcus sanguinis. Microbiol (Reading) 160:2627–2638. doi:10.1099/mic.0.082156-0 PMC4252910 25280752

[39] Zheng L, Itzek A, Chen Z, Kreth J. 2011. Environmental influences on competitive hydrogen peroxide production in Streptococcus gordonii. Appl Environ Microbiol 77:4318–4328. doi:10.1128/AEM.00309-11 21571883 PMC3127700

[40] Klementiev AD, Garg N, Whiteley M. 2024. Identification of a glutathione transporter in A. actinomycetemcomitans. Microbiol Spectr 12:e0351123. doi:10.1128/spectrum.03511-23 38051055 PMC10782972

[41] Adamowicz EM, Flynn J, Hunter RC, Harcombe WR. 2018. Cross-feeding modulates antibiotic tolerance in bacterial communities. ISME J 12:2723–2735. doi:10.1038/s41396-018-0212-z 29991761 PMC6194032

[42] Bottery MJ, Matthews JL, Wood AJ, Johansen HK, Pitchford JW, Friman VP. 2022. Inter-species interactions alter antibiotic efficacy in bacterial communities. ISME J 16:812–821. doi:10.1038/s41396-021-01130-6 34628478 PMC8857223

[43] Bottery MJ, Pitchford JW, Friman VP. 2021. Ecology and evolution of antimicrobial resistance in bacterial communities. ISME J 15:939–948. doi:10.1038/s41396-020-00832-7 33219299 PMC8115348

[44] Nadig PS, Shah MA. 2016. Tetracycline as local drug delivery in treatment of chronic periodontitis: a systematic review and meta-analysis. J Indian Soc Periodontol 20:576–583. doi:10.4103/jisp.jisp_97_17 29238136 PMC5713079

[45] Pavia M, Nobile CGA, Angelillo IF. 2003. Meta-analysis of local tetracycline in treating chronic periodontitis. J Periodontol 74:916–932. doi:10.1902/jop.2003.74.6.916 12887006

[46] Zainuddin SLA, Latib N, Taib H, Ahmad B, Sabarudin MA, Wan Mohamad WM. 2024. Effectiveness of conventional periodontal treatment with tetracycline fiber versus minocycline gel application subgingivally in periodontitis patients. Cureus 16:e55167. doi:10.7759/cureus.55167 38558744 PMC10980540

[47] Lewin GR, Stacy A, Michie KL, Lamont RJ, Whiteley M. 2019. Large-scale identification of pathogen essential genes during coinfection with sympatric and allopatric microbes. Proc Natl Acad Sci U S A 116:19685–19694. doi:10.1073/pnas.1907619116 31427504 PMC6765283

[48] Zhu B, Macleod LC, Newsome E, Liu J, Xu P. 2019. Aggregatibacter actinomycetemcomitans mediates protection of Porphyromonas gingivalis from Streptococcus sanguinis hydrogen peroxide production in multi-species biofilms. Sci Rep 9:4944. doi:10.1038/s41598-019-41467-9 30894650 PMC6426879

[49] Mintz KP, Fives-Taylor PM. 2000. impA, a gene coding for an inner membrane protein, influences colonial morphology of Actinobacillus actinomycetemcomitans. Infect Immun 68:6580–6586. doi:10.1128/IAI.68.12.6580-6586.2000 11083768 PMC97753

[50] Flyg C, Kenne K, Boman HG. 1980. Insect pathogenic properties of Serratia marcescens: phage-resistant mutants with a decreased resistance to Cecropia immunity and a decreased virulence to Drosophila. J Gen Microbiol 120:173–181. doi:10.1099/00221287-120-1-173 7012273

[51] Socransky SS, Dzink JL, Smith CM. 1985. Chemically defined medium for oral microorganisms. J Clin Microbiol 22:303–305. doi:10.1128/jcm.22.2.303-305.1985 3897273 PMC268381

