# Supplementary material for: Microbial interactions impact stress tolerance in a model oral community
Source: Microbiol Spectr. 2024 Sep 13;12(10):e01005-24. doi: 10.1128/spectrum.01005-24 (PMC11448157; doi:10.1128/spectrum.01005-24)
Supplement: Supplemental material — Fig. S1 to S3. [file spectrum.01005-24-s0001.docx]

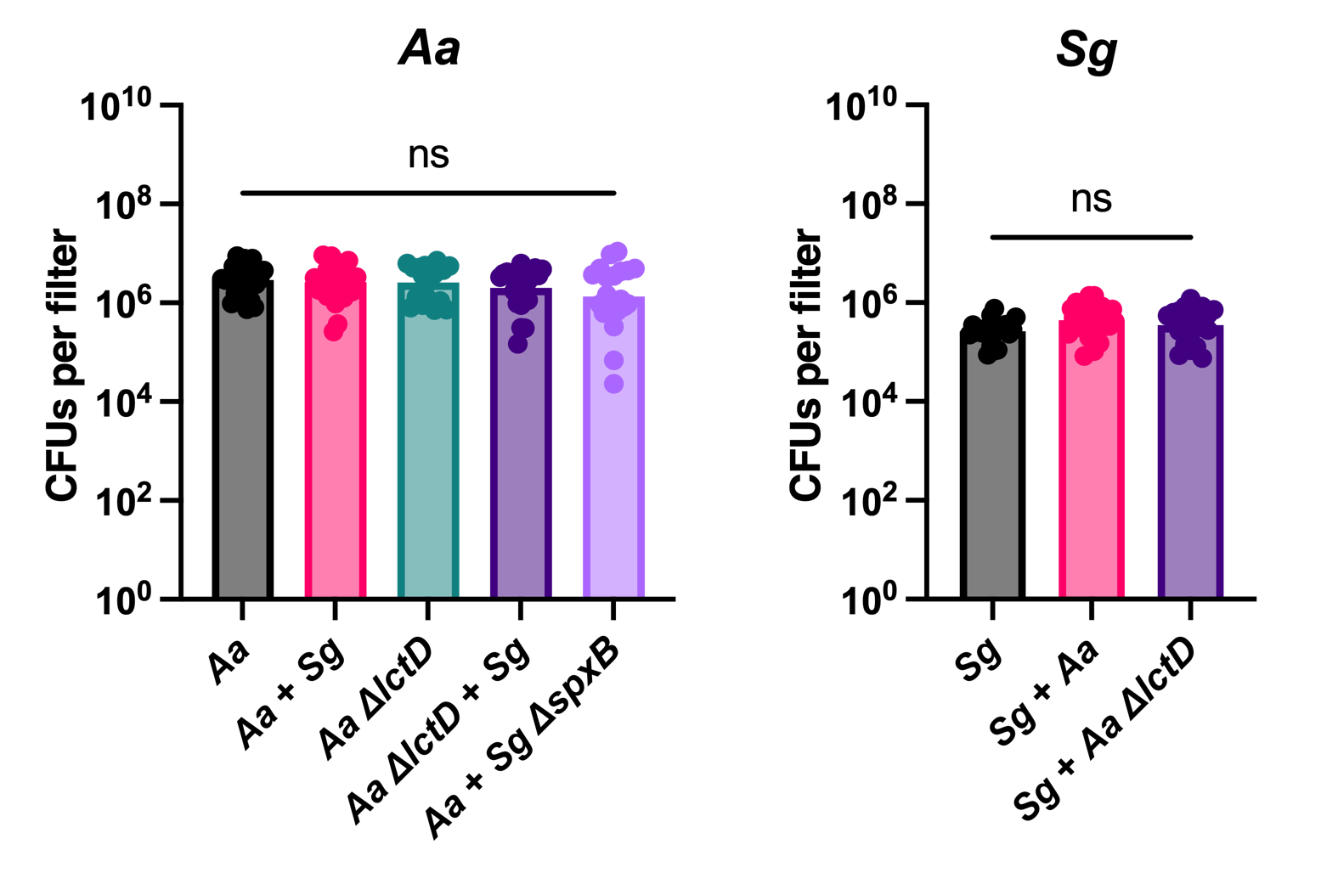


**Fig. S1. *A. actinomycetemcomitans* (*Aa*) and *S. gordonii* (*Sg*) viable cell numbers in 4-hour colony biofilms grown with and without environmental stresses.** Statistical differences were determined by one-way ANOVA.


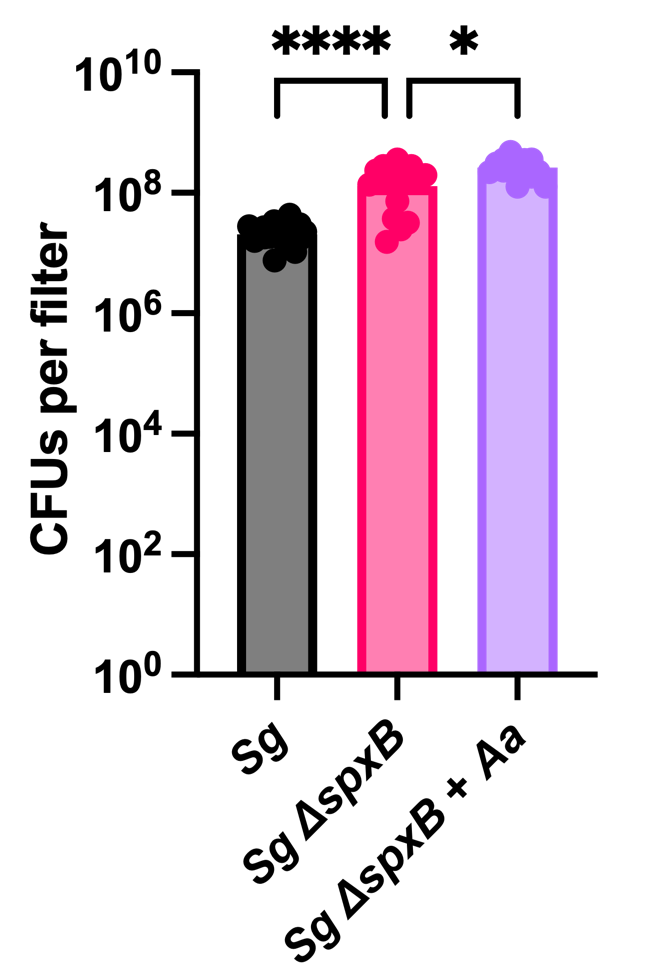


**Fig S2.** **Growth of *Sg ΔspxB* in mono- and co-culture in colony biofilms.** Statistical differences of select comparisons as determined by a Dunnett's T3 multiple comparisons test are indicated as *, *P* < 0.05; ****, *P* < 0.0001.


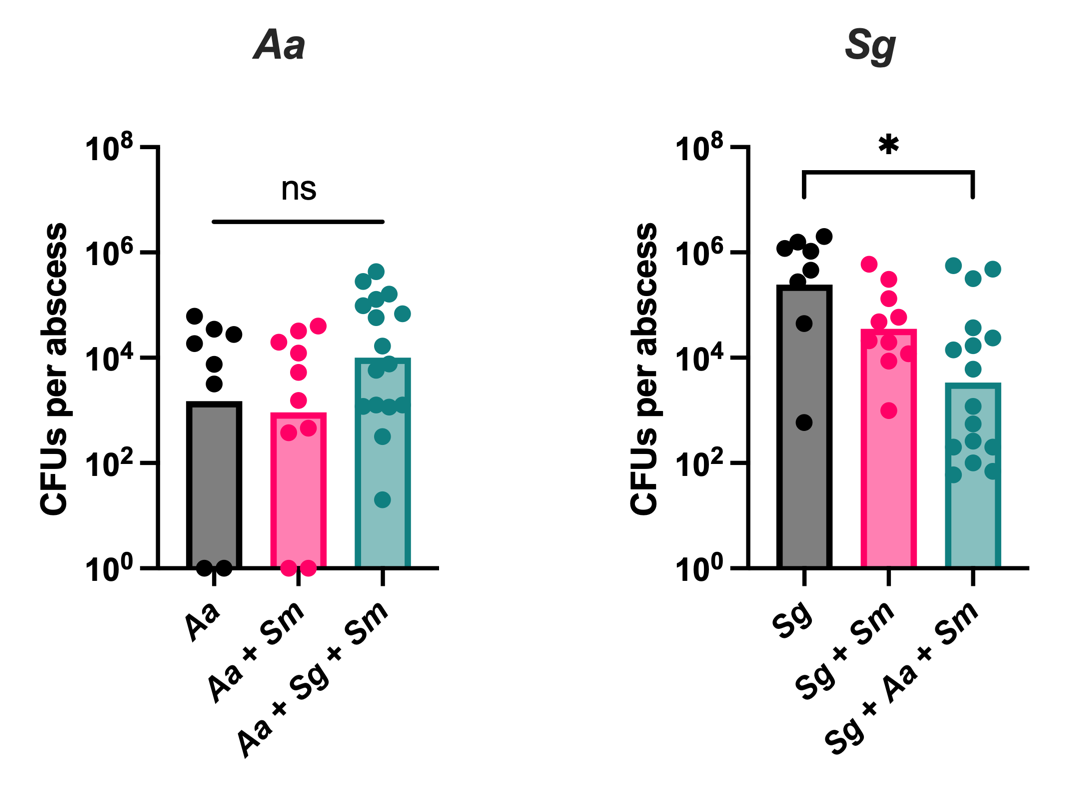


**Fig. S3. *A. actinomycetemcomitans* (*Aa*) and *S. gordonii* (*Sg*) viable cell numbers in abscess infections.** Each point is an individual abscess. Statistical differences were determined one way ANOVA and by a Dunnett's T3 multiple comparisons test. *, *P* < 0.05.
